# The COVID-19 Pandemic in the Nawalparasi District of Nepal: A Mixed Methods Assessment of Increased Alcohol Use and Intimate Partner Violence

**DOI:** 10.21203/rs.3.rs-1786122/v1

**Published:** 2022-07-14

**Authors:** Alia Cornell, Nadia Diamond-Smith, Ashley Mitchell, Mahesh Puri

**Affiliations:** University of California, Los Angeles; University of California, San Francisco; University of California, San Francisco; Center for Research on Environment Health and Population Activities

**Keywords:** COVID-19, IPV, alcohol use, households, economic insecurity, intervention

## Abstract

**Background:**

In Nepal and across the globe, the COVID-19 pandemic has primed an environment for increased rates of intimate partner violence (IPV). This paper examines how the upstream factors of alcohol use and economic insecurity in the Nawalparasi district of Nepal has brought about higher rates of IPV among newly married women.

**Methods:**

This study is a secondary analysis of data obtained from the *Sumadhur* Intervention pilot study that has been previously described and demonstrates successful implementation of group-based, household-level intervention for women’s empowerment and sexual and reproductive health education (1). Our three sets of data were collected before and during the COVID-19 pandemic. The first set is from a longitudinal cohort of 200 newly married women who were surveyed twice a year from February 2017 through July 2020. The second data set is from a cohort of newly married women, their husbands, and their mothers-in-law (31 women, 31 husbands and 31 mothers-in-law) who participated in *Sumadhur* in January 2021. The third data set was obtained through in-depth interviews in July 2021 from 15 households following *Sumadhur.* The interviews were thematically coded, and subthemes were identified. The survey data was analyzed for change over time.

**Results:**

In households in the Nawalparasi district of Nepal, between 2019 and 2020 there was an increase in alcohol consumption with reports of drinking every day increasing from 9.2% to 13.6%. In July 2020, 30% (N=31/102) of newly married women said their husbands’ alcohol consumption had increased since the pandemic. In 2019, 47.06% (N=88/187) of participants reported that they had experienced any form of IPV. In July 2021, 74% (N=23/31) of women reported being physically forced to have sexual intercourse with their husband when they did not want to and in the past four months, 68% (N=21/31) reported being forced to perform sexual acts against their will. The interviews highlighted the presence of alcohol use in the community as well as increased concerns over economic insecurity. Mothers-in-law consistently described increased rates of IPV and community violence since the pandemic.

**Conclusions:**

The pandemic has led to precarious economic situations that have influenced alcohol use among men, and instances of IPV among young, newly married women. We have demonstrated a need for urgent programmatic and policy responses aimed at reducing IPV, which has increased during the pandemic. Family interventions centering on women, such as *Sumadhur,* are critical to implement along with community-wide emergency preparedness to ensure autonomy, safety, and wellbeing now and in future times of uncertainty.

## Background

Over the past two years, the COVID-19 pandemic has created increased stressors on socioeconomic, political, and healthcare systems across the world (2). These challenges and instabilities have brought about coping strategies, such as alcohol misuse, that have heightened the opportunity for violence (3). Vulnerable groups, especially women and children, are most likely to experience the harmful ripple effects of maladapted coping strategies, increasing their risk of being of being victims of violence (4).

Intimate Partner Violence (IPV) is a worldwide public health and human rights problem exacerbated by the use of alcohol (4). IPV encompasses acts of physical violence, sexual violence, emotional or psychological abuse and controlling behaviors perpetrated by a current or former intimate partner (5). IPV is among the leading causes of death for people aged 15–44 years (6). It is estimated that 1 in 3 women experience some form of IPV in their lifetime (7,8). South Asia has one of the highest rates of IPV in the world, with an estimate of 42% of ever partnered women reporting IPV (9).

Prevention of IPV is encompassed in Goal 5 and Goal 16 of the United Nations (UN) 2030 Sustainable Development Goals (SDGs) which were initiated in 2015 after the UN Conference on Sustainable Development in Rio de Janeiro, Brazil (10). Goal 5 is “to achieve gender equality and to empower all women and girls”, which includes eliminating all forms of violence against women and girls and places an emphasis on the importance of adopting and strengthening policies and legislation to protect them (11). Goal 16 calls for promotion of peaceful and inclusive societies, to significantly reduce all forms of violence and to spread equal access justice for all (11). Combined, the 17 Goals emphasize the interconnectedness of women’s safety and empowerment for sustainable progress to a healthy and prosperous world. The Government of Nepal has internalized the SDGs to inform development plans within the country and submitted a Voluntary National Review in 2020 to the UN on progress of the goals (12).

The Government of Nepal has shown commitment to addressing the issue of violence against women including IPV specifically. Since 2009, several laws and plans of action have been enacted to prevent violence against women such as the Domestic Violence (Offence and Punishment) Act in 2009 and the National Plan of Action against gender-based violence (in 2010, and from 2013–2018). Furthermore, the new 2015 Constitution of Nepal represents a significant milestone for gender equity and social inclusion, protecting some rights for women, the poor, Gender-Based Violence (GBV) survivors and other vulnerable and marginalized groups. On August 24th 2018, the House of Representatives passed a four-point resolution on ending violence against women and created a high-level mechanism for ensuring its implementation. However, a study mapping institutional, legal and policy responses for addressing GBV revealed many challenges that remain, particularly in enforcing the laws and implementing the policies and programs. Even amidst an adaptive political scene, studies have shown that IPV is still widespread in Nepal (13). Women in Nepal are more likely than global averages to experience violence in their households, with estimates of IPV between 25–33% of ever partnered women in urban settings and up to 50% in rural areas (4,14–16).

While not yet quantified in Nepal specifically, since the COVID-19 pandemic the UN has identified exponentially rising cases of IPV across the globe (17). Home confinement measures that increase contact with a perpetrator and reduce the ability to report violence in a safe and concealed manner are considerable causes (3,18). This paper speculates that there are also upstream factors including economic insecurity and increased alcohol use that have been instigated by the pandemic and have contributed to the surge in violence.

In Nepal, alcohol use is socially tolerable among most social groups (19) and country-wide increases in alcohol use have occurred over the past few decades (19,20). In Nepal, and around the world, alcohol consumption and binge drinking is significantly higher among men than women with more than a quarter of men in Nepal experiencing alcohol dependence (4,20). Alcohol use is a predictor of aggressive and violent behavior, and women have a higher chance of experiencing sexual violence if their husband uses alcohol (5,16,21–23) Additionally, IPV has been found to be more severe when alcohol use is involved (4).

Isolation and stress, common dynamics of the pandemic, have been correlated to rises in alcohol consumption (4,22–24). Since the pandemic, there has also been limited support for those suffering from an alcohol use disorder due to the overextension of healthcare systems and reduced mobility, especially in rural communities (4,23,24). The increased use of alcohol as a result of the pandemic is predicted to cause a rise in IPV to correlate to the rise in alcohol use (23,24). Thus far, there is no evidence about how the COVID-19 pandemic has impacted alcohol use in Nepal, nor if its use has been associated with increased IPV.

The pandemic has also produced an economic downturn, which has exacerbated challenges for already vulnerable communities (25). In Nepal, 62.3% of those employed work in the informal sector which, in the wake of the pandemic, has left large portions of the population jobless and without social protections (18). Economic insecurity at the household level has been correlated to substance abuse and various forms of conflict and IPV globally (3,26). A recent study, conducted with the same research team and in the same location as this study, showed that increases in IPV during the pandemic differed by level of food insecurity, a marker of economic stress (25). Understanding if the economic downturn in Nepal has led to increases of violence can inform the impact of the COVID-19 pandemic on communities and specifically the women in these communities (25).

Women, and especially newly married women, have the lowest levels of autonomy in their household in Nepal (27). They are highly vulnerable to the ramifications of alcohol use because they are likely to experience violence from their husband as well as their in-laws (4,21,27,28). Despite the role that household members play in IPV and the fact that IPV happens at the household level, few studies capture the perspectives of family members when seeking to understand more about causes and consequences of IPV. In this study, we explore how young, newly married women were impacted by pandemic-driven changes in socio-economic status, alcohol use and IPV using data from a longitudinal sample of young, newly married women in rural Nepal, combined with qualitative data collected during the pandemic with newly married women, their husbands, and mothers-in-law in the same communities.

## Data And Methods

Data from this paper are from a larger study in collaboration with The Center for Research on Environment Health and Population Activities (CREHPA), Vijaya Development Resource Center (VDRC-Nepal), and the University of California San Francisco (UCSF). The primary topics of focus for the parent study were preconception nutrition, reproductive health, and women’s empowerment, with a focus on household harmony between newly married women, their husbands, and their mothers-in-law. Thorough methods have been published elsewhere (1, 29). This paper pulls data from three phases of the study, all of which took place in the Nawalparsi district of Nepal, near the Indian border. This district was selected for its relatively low indicators of women’s status and empowerment. The multiple phases of data collection are delineated below, as well as summarized in Table 1.

### Phase 1:

Between 2018–2020, our binational team conducted a two and-a-half-year longitudinal study with 200 newly married women termed the Formative Longitudinal Cohort Study. The women were surveyed every 6–9 months from February 2017 to July 2020 (total of 5 rounds of data collection). Women were between the ages of 18–25 at the start of the study and had been married in the last 4 months. More details about study design, recruitment, data collection, and initial findings are published elsewhere (1,29). Based on this formative research, our team developed *Sumadhur* (meaning “Best Relationship”), an interactive group-based intervention that focused on reproductive health education, gender-based discrimination, violence prevention and nutrition education for newly married women, their husbands, and their mothers-in-law. The intervention design was informed by data from the first 4 rounds of the Formative Longitudinal Cohort Study (2017–2019). The data collection in July 2020 was an extra round added to gauge the effects of the pandemic four months after a nationwide lockdown and did not inform the initial development of the intervention as it was already underway. The *Sumadhur* intervention was pilot tested between 2020–2021 among newly married women and their households (total 93 participants, 31 each of newly married women, their husbands, and mothers-in-law) (29). Most of these participants had previously been survey respondents throughout the first four rounds of the Formative Longitudinal Cohort Study. Only two women that were approached for the Formative Longitudinal Cohort Study opted to not participate in the study.

### Phase 2 (simultaneous):

Amidst the pilot study of *Sumadhur* in January 2021, another survey was designed and administered to investigate the effects of the pandemic specifically. This survey was administered to all of the 31 household groups that were participating in the *Sumadhur* intervention.

### Phase 3 (simultaneous):

Upon completion of the *Sumadhur* intervention, in July 2021 the local research team conducted in-depth qualitative interviews with 15 households (45 interviews total) who had participated in the intervention.

Throughout all phases of the study, participants provided written consent (thumb print if illiterate), including consent for the in-depth interviews to be audio recorded. All surveys and interviews were held in person in a private space in participants’ homes at a time of their choosing.

## Analysis:

### Quantitative:

Quantitative data was cleaned, coded, and summarized using STATA version 15. Exported tables were analyzed for percent change over time (30). Graphs were developed on Microsoft Excel version 16.62 (31).

### Qualitative:

Qualitative data was analyzed using organized thematic analysis (32). The interviews were first translated from the local language and then preliminarily reviewed to inform the development of a codebook. The codebook organized topics from the interview guide and topics that commonly arose in conversation without prompt. A quarter of the interviews were joint-coded by a team of three (NDS, ADC, AM) in an iterative process using the software Dedoose Version 9.0.17 (2021) (33). During this process, adjustments to the codebook were made to ensure consistency and coverage of themes. Upon completion of coding all transcripts, coded text was exported to be analyzed. The present study focused on 3 codes relevant to the study: alcohol use, IPV, and COVID-19. Qualitative data were analyzed by household to assess for patterns and changes over time.

## Survey Measures:

### Alcohol consumption:

In Phase 1, the Formative Longitudinal Cohort Study, the husband’s alcohol consumption was measured by asking their wife “How often does your husband drink any type of alcohol?” which included the response options of rarely (< once a month), sometimes (one or two times a month), often (at least once a week), very often (everyday) or don’t know. In the last round of the Formative Longitudinal Study (3 months into the COVID-19 pandemic), an additional question was asked to the women, “Has there been any changes in your husband’s alcohol consumption in the last three months?” with the response options of yes or no.

### IPV:

In Phase 1, the Formative Longitudinal Cohort Study, IPV was measured by asking the women, “Have you faced any violence from an intimate partner?” with the possible responses of yes or no. In Phase 2, the Quantitative COVID Study, IPV was measured with the prompt, “In the last four months has your husband…” with a list of various forms of abuse from which the woman respondents could select multiple.

### COVID-19 impact:

Questions about the impact of COVID-19 were collected in the July 2020 round of data from Phase 1, the Formative Longitudinal Cohort Study. Women were asked a question about the impact of COVID with options to select all that applied. Answer options included if there had been an increase in household conflict due to the pandemic and if someone in their household had lost income due to the pandemic.

All phases of this study received ethical approval from the University for California, San Francisco, and the Nepal Health Research Council (Reg No 385/2016).

## Results

### Quantitative

**Figure 1:**
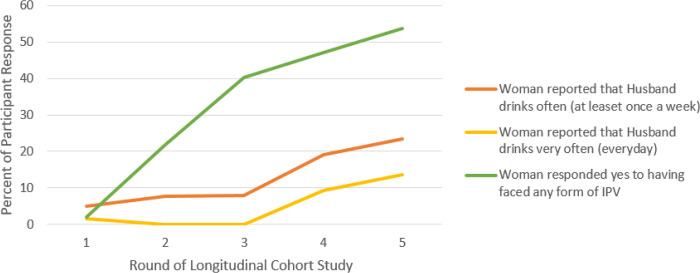
Correlation Between Alcohol Use Frequency and IPV Across Longitudinal Cohort Study Round 1 (February 2017) to Round 5 (July 2020)

As seen in Figure 1, the Formative Longitudinal Cohort Study showed an overall increase in alcohol use between February 2017- July 2020. Drinking “often (at least one time a week)” increased from 5% (N=10/200) at baseline (round 1, 2018) to 23.4% (N=43/184) (round 5, 2020). Drinking “very often (everyday)” increased from 1.5% (N=3/200) at baseline (round 1, 2018) to 13.6% (N=25/184) (round 5, 2020). After the onset of the pandemic specifically, between 2019–2020 (round 4, 2019 to round 5, 2020) drinking “often (at least one time a week)” and “very often (everyday)” both increased 4.4% with “often (at least one time a week)” increasing from 19% (N=35) to 23.4% (N=43) and drinking “very often (everyday)” increasing from 9.2% (N=17) to 13.6% (N=25).

To asses the influence of the pandemic on alcohol consumption and economic insecurity, Table 2 was compiled to display the results of Round 5 of Phase 1: the Formative Longitudinal Cohort Study (July 2020). This table shows that 30% (N=31/184) of newly married women said their husband’s alcohol consumption in the past three months (in the beginning of the pandemic) had increased. Additionally, 55.9% (N=105/184) reported that there had been changes in household conflict due to the pandemic. Most (83.5%, N=157/184) also reported that someone in their household had lost income due to the pandemic and 41.5% (N=78/184) expressed that “not being able to meet basic needs of food and shelter” was the top challenge they were facing due to the pandemic. Thirty-three out of 184 newly married women (17.9%) ranked “worrying that someone in the family or home will use violence against me” within their top five top concerns out of 12 concerns total.

In Phase 2: the Quantitative COVID Study (a year into the COVID-19 pandemic in January 2021), 74% (N=23/31) of women reported being physically forced to have sexual intercourse with their husband when they did not want to and 68% (N=21/31) reported being forced to perform sexual acts they did not want to in the past four months. This can be compared to Phase 1: the Formative Longitudinal Cohort Study from which women in 2019 had 47.06% (N=88/187) reported that they had ever experienced any form of IPV. Additionally, from Phase 2: the COVID Study Part 1, 42% (N=13/31) of women reported that they were prohibited by their husband from getting a job, going to work, trading or earning money in the past four months.

### Qualitative

The qualitative data also revealed increased rates of alcohol use and IPV due to the COVID-19 pandemic. Uniquely, there was mention of increases in general community violence. Mother-in-laws discussed the ways that the COVID-19 pandemic increased family contact and aggravation:

“During this time of Corona, people fear each other. There have been lockdowns for long periods of time. Many people don’t have a job. As all the [family] members stay at home all day, there are fights between family members for small issues. There are different types of people in the community. Some are aggressive and quarrel with family members. There has not been any kind of fights and misunderstanding in my family due to covid. But there has been violence in my neighborhood. There have been fights and quarrels among family members in their house”.

(Mother-in-law #13, age 48).

In the interviews, reference to “violence in my neighborhood” may have indicated instances of IPV or may have generally referred to disruptions and quarrels between neighbors or family members.

Other participants commonly discussed the financial impacts of the pandemic and what they perceived to be the resulting challenging consequences. A mother-in-law and a newly married woman discussed how the economic hardship of being jobless led to increased rates of alcohol use and conflict in the community:

“It’s closed everywhere. The males of the house haven’t been able to go out of the house. If there’s no job, then there’s no money. And if there’s no money, it will be hard for food and clothing. That’s why many conflicts are happening. When there’s no work to do, many kinds of thoughts come to mind. Therefore, different kinds of incidents are happening in society like theft, abuse, drinking alcohol, fighting in the society, and gambling. These kinds of incidents are heard to be happening in the society. if the person had a job to do, then they would focus on that. When they have no work, bad thoughts and opinions come to mind, because of which the society gets badly affected.”

(Mother-in-law #9, age 54).

“Because of Covid-19, people don’t have any jobs and as they don’t have jobs it is difficult to even eat. When people don’t have enough to eat or wear, there will be fights at home, psychological distress increases, and this can be been seen in many households. My father-in-law is constantly complaining about how there is no business. When people do not have enough money to buy food, the fights and violence increases within the family”.

(Newly Married Woman #14, age 20).

The pandemic was described to have a compounding effect on the lives of the poor and on the community’s youth. The lockdown created an atmosphere that increased the risk of alcohol use and misuse among these groups in particular:

This corona has ruined the life of poor people. Everyone has lost their job. There is no money if there’s no job. Lack of money has led to shortage of food. The boys of the village are seen to be drinking, gambling, and fighting as they are free the whole day. There has been a lot of stress due to corona.

(Mother-in-Law #5, age 41).

The mother-in-law group were the most likely to discuss the impacts of the pandemic and point out increased IPV in the community. Many husbands expressed that COVID-19 had not had a negative impact on the community, or they were likely to downplay the extent of the violence:

“There wasn’t any violence due to COVID-19 in my household. Due to COVID lockdown, people could not work which reduced the household income. This led to some disputes in some households of the community. It was not too big though. It was just normal household disputes. For example, husband couldn’t go out to work so he drank alcohol in a nearby place. Upon returning home he started shouting to the family members and it created dispute.”

(Husband #30, age 27).

Some husbands, however, did express that IPV was commonly observed in their communities. They noted that violence most often takes place in households when the husband or father-in-law is under the influence of alcohol. The frequency of alcohol use and IPV was associated with lower class families:

“Yes, [in my community] there are cases of violence. This has reduced though. This usually happens in low-income families where husbands come home drunk and start beating their wives.”

(Husband #9, age 26).

“Participant: Many people stayed home during the lockdown, they went through financial scarcity. This gave rise to violence. Due to lack of money, there were disputes leading to violence.

Interviewer: How often was it seen?

Participant: It was frequently observed. Violence was mostly seen in the houses where husband is an alcoholic.”

(Husband #13, age 22).

## Discussion

The results bring stark attention to the interplay between alcohol use, economic struggle, and violence against young, newly married women in the wake of the COVID-19 pandemic in Nepal. The survey data showed a longitudinal increase in alcohol consumption between 2019 and the onset of the pandemic in 2020. Across this same time period, there was a 50% increase in newly married women reporting IPV, which is consistent with other studies that show a direct correlation between IPV and alcohol use (4,14,21,34,35). The economic hardship that was reported, and the high percentages of women (42%) that reported being prohibited from getting a job or trading to earn money, reiterates the lack of autonomy that women have in their households and suggests that gender barriers have compounded the economic stressors that have led to cascades of violence (18,27).

The qualitative data give voice to the household unit and show the community-level impact of the pandemic. There is repeated concern over the increase in economic hardship, alcohol use and violence, with one factor incurring the next. In these communities, the impacts of the lockdowns have led to fears of neighbors, loss of jobs, and a more prominent atmosphere of public disruption and alcohol use. Mothers-in-law were more likely than both newly married women and husbands to speak out about these changes in alcohol use and violence prevention since the pandemic. This may have been because mothers-in-law are more sociable with other households, whereas the newly married women are often restricted to staying in their home. The husbands were the most likely participants to express little to minimal rise in alcohol use in violence since the pandemic, possibly for fear of vindicating themselves. This also highlights the frequency of mothers-in-law and newly married women as subjects of violence and supports global trends of higher alcohol use among men than women, and higher rates of violence against women than men (20,34).

The results align with previous findings that alcohol consumption rises during times of crisis, uncertainty, and economic stress (3,23,24,34). Our data underscore the critical need for mitigation efforts to address these intersecting factors. The implementation of interactive, group-based interventions like *Sumadhur,* could increase community and household-level knowledge surrounding gender equality, IPV prevention and country-specific laws on the safety and protection of women. Parallel interventions targeting husbands and fathers-in-law could specifically address alcohol use as a tangential method of decreasing rates of IPV (4,19,20,27,34,35). This study also suggests that greater access to support hotlines, reporting services and healthcare for victims of violence may be necessary in rural and under resourced areas, especially during times of increased hardship (2).

In Nepal, targeting persistent and constrictive gender norms is another important avenue for change. It is important that program intervention especially for young newly married couples address the wider societal basis of gender-based discrimination and violence. Educating on an equal worldview within programs designed to address the self-image of newly married women, their own expectations of their role as well as expectations of their spouses, families, and children, could be greatly beneficial.

### Limitations:

Our data is unique in that it collects longitudinal data both prior to and during the COVID-19 pandemic, giving insight into how the lives of people changed due to the pandemic. It combines longitudinal data with triadic qualitative data, adding richness and multiple voices to our understanding of the interplay between COVID-19, alcohol use, and IPV. Despite these strengths, this study has four main limitations. Data was collected from one district of Nepal, thus limiting the generalizability of the findings to other parts of Nepal or other countries. Not all participants were involved in every phase of data collection, and some of the participants from Phase 1: The Longitudinal Cohort Study did not participate in Phases 2 and 3 of data collection as they were a subset of this cohort. Additionally, the timeline of data collection was disrupted by the pandemic, and some of the quantitative and qualitative rounds of data were collected up to one year apart (between July 2020-July 2021). Thus, there may be more differences between the experiences in the two samples, although they were both newly married women (and their households) and were from the same district of Nepal. Although we have longitudinal data, we were unable to establish causal associations between alcohol, IPV and COVID-19. The qualitative data as well as the timing of COVID-19 within the data collection period, sheds light on the direction of association between these factors, but the small sample sizes limited the use of conducting regression analyses to establish a tighter association. Finally, alcohol use and IPV were not the main focus of the parent study, and thus we are limited in the detail of the data about these experiences. Future research focusing on causes and level of increase alcohol consumption, the link to IPV and interventions that can reduce IPV in times of social crisis, such as COVID-19 is urgently needed.

## Conclusion

Progress that has been made on gender equality and IPV prevention in the past few decades has been threatened by the COVID-19 pandemic. In Nepal, alcohol use and economic insecurity has increased, with rates of household conflict and IPV also increasing. Family interventions that center on women, like *Sumadhur,* are critical along with community-wide emergency preparedness to ensure autonomy, safety, and wellbeing in future times of uncertainty in Nepal and in other regions of the world. The findings from this study underscore urges from the UN that women should be at the center of recovery efforts from the pandemic (36). Community-based capacity building through women’s empowerment initiatives and policy to protect the safety of women should be high priority to reach the UN SGDs 5 and 16 and create healthier, safer, and more equitable societies.

## Figures and Tables

**Table 1:** Phases of Qualitative and Quantitative Data Collection

Study Phase and Reference Name	Year(s)	Population	Type of study	Frequency
Phase 1: Formative Longitudinal Cohort Study	February 2017-July 2020	200 Newly married women (age 18–25)	Survey	5 rounds, every 6–9 months.
Phase 2 (simultaneous): Quantitative COVID Study	January 2021	31 newly married women (age 18–25), 31 of their husbands and 31 of their mothers-in-law	Survey	Once
Phase 3 (simultaneous): Qualitative COVID Study	July 2021	15 newly married women (age 18–25), 15 of their husbands and 15 of their mothers-in-law	Qualitative interviews	Once

**Table 2:** Survey Results of COVID-Relation Questions in Formative Longitudinal Cohort Study Round 5, July 2020

Question and Answer Options	Participant Responses: Round 5, July 2020 (N=184) N (%)
Due to Coronavirus, has there been any changes in household conflict among your family?	
No	83 (44.1)
Yes	105 (55.9)
Has there been any changes in your husband’s alcohol consumption in the past three months (women only, N=102)	
Increase	31 (30.4)
Decrease	12 (11.8)
Remained same	59 (57.8)
Has anyone in your household lost income due to the COVID-19 pandemic?	
No	31 (16.5)
Yes	157 (83.5)
